# Recent Trends on the Future of Graduate Education in the Pharmaceutical Sciences and Research

**DOI:** 10.4103/0975-1483.63173

**Published:** 2010

**Authors:** KSS Kushwaha, N Kushwaha, AK Rai

**Affiliations:** *Institute of Pharmacy, Pranveer Singh Institute of Technology, Kanpur, (U.P.) India*

**Keywords:** Education, pharmacy curriculum, research

## Abstract

Harmonization of pharmacy education has to be made a global agenda that will encompass the developments that have taken place in basic, medical, pharmaceutical sciences in serving the needs and expectations of the society. The professional pharmacy curriculum is designed to produce pharmacists who have the abilities and skills to provide drug information, education, and pharmaceutical care to patients; manage the pharmacy and its medication distribution and control systems; and promote public health. Required coursework for all pharmacy students includes pharmaceutical chemistry; pharmaceutics (drug dosage forms, delivery, and disposition in the human body) pharmacology; therapeutics (the clinical use of drugs and dietary supplements in patients); drug information and analysis; pharmacy administration (including pharmacy law, bioethics, health systems, pharmacoeconomics, medical informatics); clinical skills (physical assessment, patient counseling, drug therapy monitoring for appropriate selection, dose, effect, interactions, use); and clinical pharmacy practice in pharmacies, industry, health maintenance organizations, hospital wards, and ambulatory care clinics.

## INTRODUCTION

Across the nation, people are living longer. This longevity is attributable to healthier lifestyles, a well-trained health workforce, advances in science and understanding of human health and disease, and continuing discovery of new therapies for managing acute and chronic conditions. As the population ages, however, its interaction with the health care system increases. Larger patient populations in general, and increasing numbers with chronic diseases in particular, contribute to rapidly rising demands for health providers and facilities that must stretch to meet growing needs.

Within the pharmacy workforce, evidence of this demand is seen in the dramatic increase in prescriptions written and dispensed in the United States. During the 1990s alone, the number of retail prescriptions dispensed increased by 45%, from 1.8 billion in 1992 to almost 2.9 billion in 1999. By 2005, this number is expected to increase to approximately 3.9 billion prescriptions.

Among the factors fueling this growth are development of new medications and drug therapies, identification of new uses for existing medications, increased numbers of authorized prescribers, broader insurance coverage for some medications, and direct marketing to the public by pharmaceutical companies. Not surprisingly, this growth has generated a corresponding demand for pharmacists in hospitals and clinics, as well as in retail, government, and academic settings. Because growth of the workforce has not kept pace with the demand for services – due in part to the lack of growth in educational opportunities – a nationwide pharmacist shortage has developed.

### Designing a pharmaceutical care-based curriculum

For designing a pharmaceutical care-based curriculum, several groups have recognized the need to link pharmacy practice functions and educational curricula, the most recent to undertake this task was the American Association of College of Pharmacy’s (AACP) Commission to Implement Change in Pharmaceutical Education.[1] The Commission was established to

- Define the missions of the profession, pharmacy practice and pharmacy education.
- Describe an entry level degree, curricular outcomes, curricular content and education processes.

This foundation took the form of

- A mission statement for the profession of pharmacy,
- A mission statement for the practice of pharmacy,
- A mission statement for pharmacy education,
- A description of the expected outcomes of the curriculum relating to the provision of pharmaceutical care, and
- A description of the curricular content, educational processes and assessment techniques needed to achieve the pharmaceutical care-specific educational outcomes.

The following sections briefly describe each of these elements as developed by the small working group:

### Mission statement for the profession

The mission of the profession is to accept responsibility for the drug-related needs of patients in society. These needs are met by pharmacists fulfilling their responsibilities at:

The patient-specific level through the practice of pharmacy (specifically, pharmaceutical care);

The professional level through education, research, and the development of policies and standards; and

The societal level through research, public education, and policy development.

Although this mission statement indicates that pharmacists have practice responsibilities at the patient specific level, it does not define clearly enough the parameters of these patient-specific responsibilities. Hence, the need for a practice mission statement.

### Mission statement for practice

The mission of pharmacy practice is to assure that a pharmacist meets each individual patient’s drug related needs through the delivery of pharmaceutical care.

### Mission statement for pharmaceutical education

**Figure 1:**
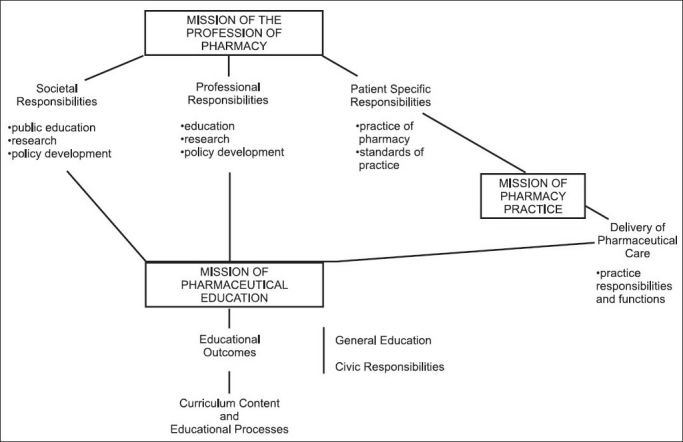
Inter-relationship of mission statements, responsibilities, educational outcomes, curricular content, and educational processes

A mission statement for pharmaceutical education was necessary to establish the link between pharmacy practice and educational outcomes [Figure 1].

The mission of pharmaceutical education is to provide a curriculum, which by its content and presentation, enables the student to learn the knowledge, skills, and values necessary to meet the drug-related needs of patients in society. This primarily occurs in practice when the pharmacist delivers pharmaceutical care to a patient, thereby meeting an individual patient’s drug-related needs. It also occurs at the professional level through education, research, and the development of policies and standards, and at the societal level through research, public education, and policy development, all directed toward the prevention of drug-related morbidity and mortality.

Functions and Responsibilities Associated with the Provision of Pharmaceutical Care

**Figure 2:**
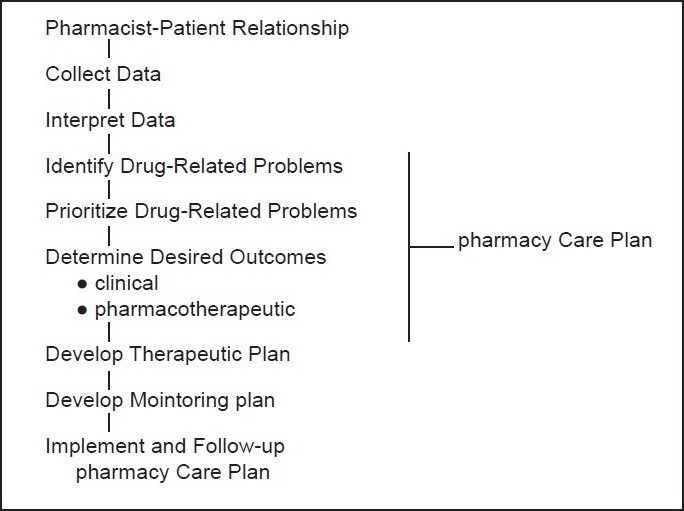
Pharmacists practice functions when providing pharmaceutical care.

Significant differences between the Commission’s work and the efforts at the University of Toronto became apparent in the list of practice functions and responsibilities developed by the two groups.[2] Figure 2 represents the work of pharmacist

Initially it was quite tempting for our working group of pharmacist functions which described the current practice of clinical pharmacy, as included in the Commission’s work.

These functions are

- Participating in the process of drug use decisions;
- Selecting the drug product dosage form;
- Determining the dose and dosage schedule;
- Preparing the drug product for patient use;
- Providing the drug product to the patient;
- Providing drug information to the patient;
- Monitoring the patient to detect adverse drug reactions and drug interactions; and
- Monitoring the patient to enhance the probability that therapy proceeds in accord with patient care objectives.

### Curricular content and educational processes

Four general principles guided the Curriculum Committee’s re-evaluation of the curriculum. They were

- The content and educational processes utilized in delivering the curriculum must directly link to the three priorities agreed to by the faculty and the broad categories of intended educational outcomes described in Table 1;
- The revised curriculum should ensure that all areas required to attain the intended educational outcomes be addressed, including knowledge, skills and values, versus the emphasis which was previously on knowledge;
- Each course included in the revised curriculum should specify the intended educational outcomes, content and educational processes to be utilized and that the course coordinator justify how these specific educational outcomes contributed toward the broad intended educational outcomes for the program; and
- Minimal repetition of material occurs through appropriate coordination and sequencing of courses.

**Table 1:** The major categories of educational outcomes associated with the three levels of professional responsibility:

Upon completion of a pharmacy curriculum, thestudent should be able to: Provide pharmaceutical care (*i.e*., patient specific responsibilities) by Correctly identifying, satisfactorily solving, and effectively preventing the drug-related problems from the eight categories which are most commonly occurring, are most likely to cause the greatest harm and are able to cause harm most quickly. Improve the profession, its organizations, and institutions (*i.e*., professional responsibilities) by Shaping policies, practices, and education Promoting the profession to other professionals and society in general Designing, conducting, and utilizing drug therapy research Better society (*i.e*., societal responsibilities) by Promoting health and well being Playing an active role in shaping health-care policies, practices, and education Ponducting research to further the knowledge needed to advance healthcare generally and medicine use specifically

The process was therefore somewhat inverted in that rather than the Curriculum Committee decreeing the specifics that must be taught in each course, course coordinators were asked to re-evaluate and then justify the educational content and processes utilized in his/her course.[3]

## BRIEF OUTLINES FOR NEW OR DSIGNIFICANTLY CHANGED COURSES

### Introduction to the profession of pharmacy (new course-year I)

This course is designed to give students a broad perspective on the profession and the practice of pharmacy. The historical background of the profession is introduced and serves as a starting point for further exploration of developments and trends in the profession and practice of pharmacy. The evolution of the practice of pharmacy (from compounding and dispensing functions to a clinical and patient care focus) is examined. The philosophy of pharmaceutical care will be introduced and developed. An important component of this course is an introduction to a variety of teaching and learning methods with particular emphasis on problem-based learning.[4]

### Professional practice (new courses - years I to IV)

#### Year I

This course is an introduction to several components of pharmacy practice, taught in a combination of large group lectures and small group workshops or tutorials. Introduction to drug information introduces the students to library and drug information storage and retrieval methods for researching and answering problems, abstracting, and referencing. Students learn to develop a systematic approach for responding to drug information requests. Professional Ethics introduces students to principles of ethics as well as a systematic approach to ethical problem-solving. Communications focuses on having students understand and develop basic verbal and non-verbal communication skills. A pharmacy practice lab in the spring requires students to effectively apply legal principles learned in jurisprudence to the processing and filling of prescriptions in simulated community pharmacies.[5]

#### Year II

This second year course continues to develop principles and skills as in Professional Practice I. The theoretical aspects of applied pharmaceutics and extemporaneous compounding form the basis of prescriptions that are used as cases in problem-based interactive sessions that coordinate with laboratories. The focus of both is the application of principles.

#### Year III

This third year course requires students to apply principles of jurisprudence, communication, group interaction, problem solving, and decision making to a variety of situations generally encountered in ambulatory pharmacy practice. Simulated pharmacies provide the environment, and a problem-based format is used where the prescription is a starting point. Pharmaceutical care is provided to patients with a focus on positive patient interaction, gathering of relevant data, identification of drug-related problems, and development of pharmacy care plans.

#### Year IV

This is a continuation of Professional Practice III. It requires students to demonstrate a consolidation of knowledge from previous Professional Practice courses and draws on and complements material from Pharmaceutical Care I, II, and III as well as other courses, in particular Health Systems and Pharmacy Management. Simulated pharmacies again provide the environment for this course. Pharmaceutical care is provided to patients, with a focus on ambulatory/community practice. Clear documentation of the provision of this care is required.

### Pharmaceutical care (new courses - years II to IV)[6]

#### Years II and III

This course is an exploration of the pharmacist’s role in self medication and focuses on the application of the pharmaceutical care process to self-care of mild and/or self-limiting conditions. The selection and use of non-prescription drugs form the basis for the development of basic principles of self-care and self-medication. Students develop a systematic approach to self-care counseling and information gathering and enhance their communication skills. Role-playing, class discussions, and workshops in this course address patients’ drug-related needs and sensitize students to the moral, ethical, and legal responsibilities related to self-care.

#### Years III and IV

Through discussion of a series of case studies taught in a problem-based format, students acquire and/or reinforce their skill at determining whether a patient’s signs or symptoms are related to drug therapy and, if so, how they are related to drug therapy and what alterations are required in the patient’s drug therapy to solve or prevent this problem. The case studies utilized reinforce relevant pathophysiological and pharmacological concepts required to make these decisions. The specific disease states discussed are common diseases that are not self-limiting. Students are expected to communicate both their decisions and the process followed in making these decisions in an understandable, appropriate written, and verbal format.

### Health systems in society (new courses - years II and IV)

#### Year II

In this small group, interactive course places the pharmacist and pharmacy practice within its societal, institutional, and professional environment. Topics include gaining a general understanding of the health-care system in Canada, the various definitions of health, how to judge the healthiness of citizens, individual action versus policy decisions, how and why care seeking occurs, and the role of the pharmacists and other care providers in the health-care system.

#### Year IV

This course builds on material covered in the Health Systems in Society I and other courses to provide a theoretical understanding of some of the social issues that concern pharmacists as well as to provide the opportunity for students to begin to formulate and express their views on specific ethical, political, and professional issues. Topics addressed include introductions to manpower issues, corporatization of pharmacy in Canada, the impact of biotechnology, care of the elderly, death and dying, alternative healthcare, health policy, health economics, and emerging trends in health-care.

### Pharmacy management (changed courses - Years III and IV)

#### Year III

This course provides the linkages between the principles of management theory and the realities of practice. Course material focuses on the organizational or institutional philosophy/practice toward material and service management which impact pharmacy practice. Focus is directed toward understanding the practice and philosophy of organizations external to pharmacy but which have a significant impact on the pharmacist’s practice.

#### Year IV

Course objectives identify the professional practice issues requiring a manager’s attention and how students, as future practitioners, can employ a management perspective to act as change agents for the profession. Repeatedly, the students are exposed to the importance of the optimal use of the profession’s human resources. The concepts of a good manager in the context of an ideal employer, an excellent place to work, and a practice goal of pharmaceutical care are emphasized.

### Pharmacy practice research (new course - year IV)

Students develop the research skills necessary to address questions commonly encountered in everyday pharmacy practice. The first series of interactive sessions allow the students to develop a step-by-step process for solving practice-related questions. The students then have the opportunity to apply this information to an assigned problem. They are responsible for developing a plan to solve this problem which they will then formally present to other students and submit as a written proposal. Students learn a range of research techniques and are required to critique the proposals outlined by other students.

### Statistics (altered course - year I)

This course introduces the basic concepts and applications of experimental design and statistical analysis in a problem-solving-based format. The main course objective is to develop the students’ ability to read the professional literature critically. To achieve this, a full range of statistical techniques are reviewed, from the simple statistics for a single variable to more complex techniques for the analysis of many variables.[7]

### Pharmaceutics (altered course - year II)

In this problem-based course, the dosage form is considered as a means of delivering a drug to the appropriate site in the appropriate concentration, for the appropriate period of time. The science of dosage form development or formulation is presented within a framework that includes economic, biopharmaceutical, patient, ethical, and quality issues.[8]

Increasing criticism of the national graduate education enterprise arose in the early 1990s, primarily from new PhD graduates in the physical sciences, who had difficulties finding desirable employment opportunities. Concerns about this “oversupply” of PhDs, or an “undersupply” of employment opportunities in the graduate’s areas of research interest began to focus national attention on graduate education. The Committee on Science, Engineering, and Public Policy (COSEPUP) of the NRC in 1995 attempted to examine the career paths of PhD graduates in the sciences and engineering, and define the most appropriate structures and functions for graduate education. COSEPUP members ”…were sufficiently troubled by the lack of generally available information to conclude that students’, professors’, and mentors’ lack of accurate, timely, and accessible data on employment trends, careers, and sources of student support is a serious flaw in our educational system.” The COSEPUP final report, “Reshaping the Graduate Education of Scientists and Engineers” concluded that “…the job opportunities of the future will favor students with greater breadth of academic and career skills…”

- The PhD degree should remain a research-intensive degree, but provide more curricular or experiential options to increase the breadth of skills of graduates.
- Potential graduate students should be provided with accurate and timely information about career prospects so students can make informed choices about their careers.
- Students have primarily been responsible for the rapid growth in the number of PhD degrees awarded in the last decade.
- The growth in PhD degrees, particularly in the biomedical sciences, has contributed to a significant increase in the number of postdoctoral fellows and time spent in postdoctoral positions.

Partly in response to growing concerns about science policy and graduate education, the COSEPUP of the NRC published a report, “Reshaping the Graduate Education of Scientists and Engineers” in 1995. This widely discussed report made a number of general recommendations as follows:

1. **Offer a broader range of academic options**
  - To produce more versatile scientists and engineers, graduate programs should provide options that allow students to gain a wider range of skills.
  - To foster versatility, government and other agents of financial assistance for graduate students should adjust their support mechanisms to include new education/training grants to institutions and departments.
  - To promote versatility, care must be taken not to compromise other important objectives when implementing changes.
2. **Provide more clear and timely information and guidance**
  - Graduate scientists and engineers and their advisers should receive more up-to-date and accurate information to help them make informed decisions about professional careers; broad electronic access to such information should be provided through a concerted nationwide effort.
  - Academic departments should provide the information referred to above to prospective and current students in a timely manner and should also provide career advice to graduate students. Students should have access to information on the full range of employment possibilities.
  - **Devise a National Human Resource Policy for Advanced Scientists and Engineers**
    A national discussion group, including representatives of governments, universities, industries, and professional organizations, should carefully examine the goals, policies, conditions, and unresolved issues of graduate-level human resources.
    These broad recommendations are aimed at improving all graduate programs in science and engineering. Although it is likely that all graduate programs and their students would benefit from these recommendations, some issues, notably those involving national funding policies.

### Status of Problem-Based Learning Research in Pharmacy Education: A Call for Future Research

Problem-based learning has been increasingly used in pharmacy education. Problem-based learning serves to enhance such skills as problem-solving, critical thinking, clinical reasoning and self-directed learning.

PBL has been incorporated into pharmacy education in an effort to prepare future pharmacists to meet the challenging demands of the pharmacy profession, in particular, the provision of quality patient care. The roots of PBL can be traced back to John Dewey. Dewey, an early educational philosopher, recommended that students should be presented with real required to solve them. Further, Dewey encouraged reflection as a process that should be used when problem solving. Dewey recognized that we can “reflect” on a whole host of things in the sense of merely “thinking about” them, however, logical or analytical reflection can take place only when there is a real problem to be solved. Both learning by doing and reflection are hallmarks of the PBL process.

In PBL, students are first presented with the patient’s presenting problem. Next, the learners engage in such clinical reasoning processes as hypothesis generation, data gathering, data analysis, and decision making, while synthesizing basic science and clinical information in an effort to offer some potential diagnoses and courses of treatment for the patient’s problem. PBL also incorporates the use of an expert tutor or facilitator who serves to guide the problem-solving process.[9]
